# Plant-derived extracellular nanovesicles in musculoskeletal repair and functional rehabilitation: mitochondrial–immune–metabolic regulation

**DOI:** 10.3389/fchem.2026.1968520

**Published:** 2026-10-12

**Authors:** Ningning Zhu, Ningrui Wang, NaNa Zhou, Baodong Ye, Jingjing Liu

**Affiliations:** 1 Department of Hematology, The First Affiliated Hospital of Zhejiang Chinese Medical University (Zhejiang Provincial Hospital of Chinese Medicine), Hangzhou, China; 2 The First School of Clinical Medicine, Zhejiang Chinese Medical University, Hangzhou, China

**Keywords:** immunometabolism, mitochondrial reprogramming, musculoskeletal regeneration, plant-derived extracellular nanovesicles, regenerative medicine, tissue repair

## Abstract

Musculoskeletal injuries and degenerative disorders caused by trauma, aging, chronic inflammation, and metabolic dysfunction frequently result in impaired functional recovery, including skeletal muscle atrophy, bone loss, cartilage degeneration, and neuromuscular dysfunction. Although exercise rehabilitation, physical therapy, and pharmacological interventions can improve clinical outcomes, their ability to restore the complex pathological microenvironment characterized by persistent inflammation, oxidative stress, mitochondrial dysfunction, and metabolic imbalance remains limited. Plant-derived extracellular nanovesicles (PELNs), naturally occurring lipid-bilayer nanoparticles containing diverse bioactive cargos such as RNAs, proteins, lipids, and phytochemicals, have recently emerged as promising regulators of tissue repair. Unlike conventional nanoparticles that mainly serve as passive delivery carriers, PELNs possess intrinsic biological activity and can actively modulate cellular states across biological barriers. Increasing evidence indicates that PELNs contribute to musculoskeletal regeneration by regulating inflammatory responses, oxidative stress, mitochondrial homeostasis, stem/progenitor cell activity, and extracellular matrix remodeling. Recent studies have reported regenerative effects of PELNs in experimental models involving skeletal muscle, bone, and peripheral nerve tissues. Beyond tissue-specific effects, accumulating evidence suggests that PELN-mediated regeneration involves coordinated mitochondrial–immune–metabolic reprogramming, including regulation of mitochondrial quality control, inflammatory signaling, and immunometabolic states. This review summarizes recent advances in PELN-mediated musculoskeletal repair and their potential relevance to functional recovery, highlighting their biological properties, therapeutic applications, and underlying regulatory mechanisms. The challenges and future perspectives of engineered PELNs for regenerative rehabilitation are also discussed.

## 1 Introduction

The musculoskeletal system is a highly integrated biological network responsible for locomotion, mechanical support, and maintenance of physical function (Zhang et al., 2019; Harkins et al., 2023). The recovery of musculoskeletal function after injury or degeneration requires coordinated regulation of multiple biological processes, including inflammatory resolution, metabolic adaptation, mitochondrial remodeling, and tissue regeneration (Hotamisligil, 2006; Rahman and Quadrilatero, 2021; Alway et al., 2023). However, a wide range of pathological conditions, including traumatic injury, aging, chronic inflammation, and metabolic disorders, can disrupt these regulatory networks and result in persistent functional impairment (Frontera and Ochala, 2015; Tidball, 2017).

Musculoskeletal dysfunction is commonly observed in skeletal muscle atrophy, sarcopenia, bone loss, cartilage degeneration, and neuromuscular disorders (Barnett, 2018; Compston et al., 2019; Cruz-Jentoft et al., 2019). Although these conditions differ in their initiating causes, they share several pathological characteristics, including excessive inflammatory activation, oxidative stress accumulation, mitochondrial dysfunction, and impaired regenerative capacity of resident stem and progenitor cells (Tidball, 2017; Rahman and Quadrilatero, 2021; Schieber and Chandel, 2014; Yin et al., 2013). These abnormalities contribute to deterioration of tissue homeostasis and create a microenvironment that restricts effective repair and functional recovery.

Current rehabilitation strategies, including exercise therapy, physical interventions, nutritional support, and pharmacological treatments, have demonstrated beneficial effects in improving functional outcomes (Giraldo-Vallejo et al., 2023; Arora et al., 2024; Rawat et al., 2026). Nevertheless, these approaches often have limited capacity to simultaneously restore the complex molecular networks involved in tissue repair. In particular, inflammation, oxidative stress, mitochondrial dysfunction, and metabolic dysregulation are highly interconnected processes rather than isolated pathological events. Therefore, therapeutic strategies capable of coordinately regulating multiple biological pathways may provide new opportunities for enhancing tissue recovery.

Conventional nanomedicine has historically been developed primarily under a passive drug-delivery paradigm, in which nanoparticles function mainly as carriers to improve drug stability, biodistribution, and therapeutic efficiency (Mitchell et al., 2021). However, for regenerative disorders involving dynamic changes in cellular states and tissue microenvironments, simple cargo delivery may be insufficient. Increasing attention has therefore shifted toward biologically active nanovesicles capable of directly regulating cellular function and restoring tissue homeostasis (Wiklander et al., 2019; Herrmann et al., 2021).

Plant-derived extracellular nanovesicles (PELNs) have recently emerged as promising bioactive nanotherapeutic systems with intrinsic regulatory properties (Gao et al., 2026). Derived from edible and medicinal plants, PELNs contain diverse functional cargos, including microRNAs, proteins, lipids, and phytochemicals, which can influence mammalian cellular behaviors through interspecies communication (Mu et al., 2014; Yu et al., 2026). Compared with synthetic nanoparticles and mammalian extracellular vesicles, PELNs exhibit several advantages, including good biocompatibility, low immunogenicity, structural stability, abundant sources, and potential for large-scale production (Dad et al., 2021).

Accumulating evidence indicates that PELNs can influence multiple biological processes associated with tissue repair, including inflammatory responses, oxidative stress control, mitochondrial homeostasis, stem cell activation, and extracellular matrix remodeling (Lu et al., 2026). In skeletal muscle, plant-derived nanovesicles have been shown to alleviate muscle atrophy and enhance regeneration through metabolic regulation and mitochondrial remodeling (Zhou et al., 2024). Similarly, PELNs have demonstrated regulatory effects in bone and cartilage repair by modulating osteogenic differentiation, extracellular matrix metabolism, and inflammatory microenvironments (Seo et al., 2023; Rashidi et al., 2025; Wang et al., 2026; Zhang C. et al., 2026).

Beyond tissue-specific effects, emerging studies suggest that the regenerative activity of PELNs may arise from their ability to coordinate mitochondrial, immune, and metabolic networks. Mitochondria serve as central regulators of cellular energy production, oxidative stress responses, and cell fate decisions (Spinelli and Haigis, 2018), while immune and metabolic pathways determine whether injured tissues remain in a degenerative state or transition toward repair (Eming et al., 2017). Through modulation of mitochondrial function, inflammatory signaling, and immunometabolic states, PELNs may facilitate the reconstruction of a regenerative microenvironment across musculoskeletal tissues (Fan et al., 2022).

In this review, we summarize recent advances in the biological characteristics and therapeutic applications of PELNs in musculoskeletal repair and their potential relevance to functional recovery (Figure 1). We further discuss their roles in mitochondrial regulation and immune–metabolic reprogramming, as well as current challenges and future perspectives for developing PELN-based regenerative rehabilitation strategies.

**FIGURE 1:**
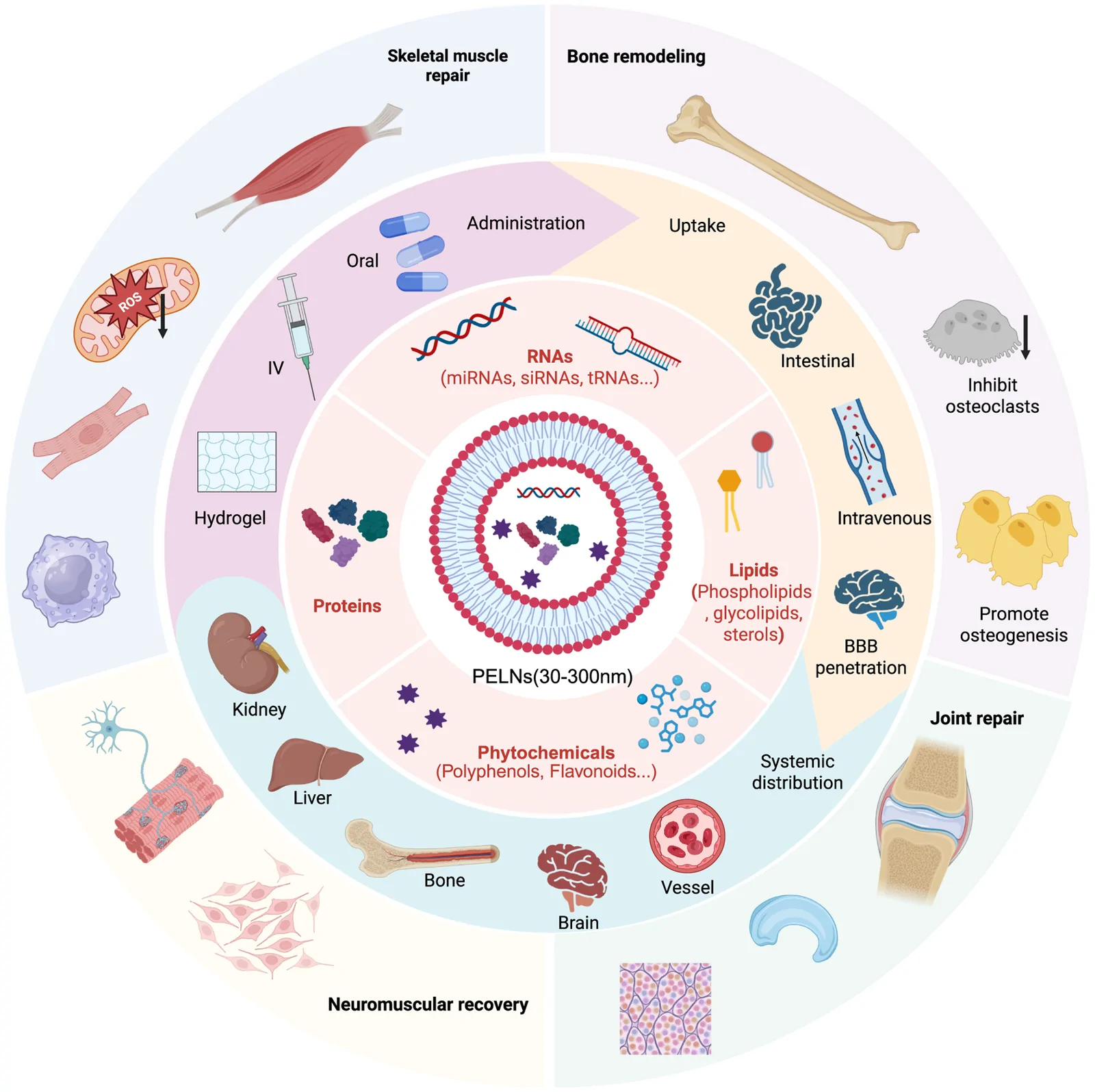
Schematic overview of the biological characteristics and regenerative mechanisms of PELNs in musculoskeletal rehabilitation. PELNs are nanosized extracellular vesicles (approximately 30–300 nm) derived from edible and medicinal plants. They contain multiple endogenous bioactive cargos, including RNAs, proteins, lipids, and phytochemicals. Following oral or systemic administration, PELNs are internalized by recipient cells and distributed to multiple tissues, where they mediate cross-kingdom biological communication. Current evidence suggests that PELNs promote musculoskeletal regeneration by coordinately regulating inflammation, oxidative stress, mitochondrial homeostasis, immune–metabolic adaptation, and intercellular communication. Through these integrated mechanisms, PELNs facilitate skeletal muscle repair, bone regeneration, cartilage and joint repair, and peripheral nerve regeneration, thereby supporting functional recovery and regenerative rehabilitation.

## 2 Biological characteristics of PELNs

Plants release nano-sized extracellular vesicles into the extracellular environment to mediate cell-to-cell communication and respond to environmental stimuli (Woith et al., 2019). These vesicles share several structural features with mammalian exosomes, including lipid bilayer membranes and nanoscale size distribution (typically 30–200 nm), and are therefore referred to as PELNs (Pinedo et al., 2021). PELNs have been isolated from various plant sources, including: fruits (grape, grapefruit, lemon), vegetables (ginger, broccoli), medicinal plants (ginseng, turmeric, herbal medicines) (Wang et al., 2024; Zeng et al., 2024). They are emerging as evolutionarily conserved nanoscale carriers that mediate interspecies communication and bioactive molecule transfer (Liu et al., 2026). Their intrinsic lipid bilayer architecture, coupled with selective cargo packaging, enables them to participate in stress adaptation in plants while simultaneously exerting regulatory effects in mammalian systems. Recent advances have further transformed PELNs from naturally occurring vesicles into engineerable nanoplatforms for precision regenerative medicine (Yu et al., 2026).

### 2.1 Biogenesis and molecular characteristics of PELNs

PELNs are nanoscale lipid bilayer vesicles naturally secreted by plant cells and have recently emerged as an important class of bioactive nanomaterials for cross-kingdom communication and regenerative medicine. Similar to mammalian extracellular vesicles (EVs), PELNs encapsulate diverse endogenous cargos within membrane-bound structures, thereby protecting biologically active molecules from degradation during extracellular transport.

Although the biogenesis of mammalian EVs has been extensively characterized, the mechanisms governing PELN formation remain less well understood. Current evidence suggests that plant nanovesicles originate from multiple intracellular membrane trafficking pathways rather than a single biosynthetic route. Vesicles may be generated through multivesicular bodies (MVBs), exocyst-positive organelles (EXPOs), vacuolar trafficking pathways, and plasma membrane budding, followed by secretion into the apoplastic space (Zeng et al., 2024). These distinct biogenetic pathways are believed to contribute to the heterogeneity of PELNs with respect to their size, membrane composition, and molecular cargos. However, compared with mammalian EVs, definitive molecular markers for individual PELN subpopulations have not yet been established, and the relationship between vesicle origin and biological function remains an active area of investigation (Pinedo et al., 2021).

Unlike synthetic nanoparticles, whose biological activities primarily depend on externally loaded therapeutic agents, PELNs possess intrinsic biological functions arising from their endogenous molecular composition (Langellotto et al., 2025). Accumulating evidence indicates that PELNs contain a diverse repertoire of small RNAs, proteins, lipids, enzymes, metabolites, and phytochemicals, many of which retain biological activity after uptake by mammalian cells (Zeng et al., 2024). Although the relative contribution of individual cargo components remains incompletely understood, these molecules are thought to act cooperatively to regulate cellular signaling, inflammatory responses, and metabolic adaptation rather than functioning independently (Jo et al., 2025). This intrinsic bioactivity distinguishes PELNs from conventional nanocarriers and provides the biological basis for their regenerative potential (Hao et al., 2024).

An additional advantage of PELNs lies in their favorable pharmaceutical and translational properties. Compared with mammalian extracellular vesicles, PELNs can be readily isolated from edible fruits, vegetables, and medicinal plants using scalable, cost-effective production procedures, while avoiding many of the biosafety, pathogen transmission, and ethical concerns associated with animal-derived materials (Langellotto et al., 2025). Moreover, accumulating evidence suggests that PELNs exhibit remarkable physicochemical stability and can maintain structural integrity under simulated gastrointestinal conditions and following oral administration in preclinical models, enabling interactions with intestinal epithelial cells, resident immune cells, and, in some cases, extra-intestinal tissues after absorption (Guan et al., 2026; Huang et al., 2026). These unique characteristics, together with their intrinsic bioactivity and favorable safety profile, make PELNs particularly attractive candidates for long-term treatment of chronic diseases and regenerative rehabilitation.

Terminology for plant-derived vesicular nanoparticles remains inconsistent across the literature. Terms such as plant-derived extracellular vesicles (PDEVs), plant-derived extracellular nanovesicles (PDENs), exosome-like nanoparticles (ELNs), and plant-derived exosome-like nanoparticles are often used for vesicular structures isolated from plant tissues or fluids. However, the term “exosome” implies a specific endosomal biogenesis pathway that has not been established for most plant-derived vesicles. In this review, we therefore use “plant-derived extracellular nanovesicles” (PELNs) as an operational umbrella term for naturally occurring, membrane-bound nanoscale vesicles isolated from plant sources, unless the terminology used in the original study is needed to identify a specific vesicle preparation. This usage does not imply a shared biogenesis pathway or biological equivalence among vesicles from different plant species.

### 2.2 Molecular composition and bioactive cargos

The biological activities of PELNs have been associated with their diverse molecular cargos, including small RNAs, proteins, lipids, enzymes, and plant-derived metabolites. Unlike synthetic nanoparticles, whose biological effects generally depend on externally incorporated cargos, PELNs contain endogenous molecules packaged within membrane-bound vesicular structures. These components may contribute to cellular signaling, inflammatory regulation, redox responses, and metabolic homeostasis after interaction with mammalian recipient cells (Langellotto et al., 2025). However, identification of a molecule within PELNs does not establish that it is responsible for a particular biological effect, and direct cargo–function relationships remain insufficiently defined for most PELN systems.

Among these cargos, small RNAs, particularly microRNAs (miRNAs), have received considerable attention because of their proposed role in cross-kingdom communication. Several studies have detected plant-derived small RNAs in vesicular preparations and suggested that they may remain stable following uptake by mammalian cells (Zhang et al., 2012). However, whether endogenous plant miRNAs directly regulate mammalian target genes under physiologically relevant conditions remains controversial. Major concerns include the specificity of predicted plant miRNA–mammalian target interactions, technical difficulties in reliably detecting very low-abundance plant miRNAs in mammalian samples, and the possibility of contamination or analytical artifacts during RNA extraction, amplification, or sequencing. In addition, it remains uncertain whether dietary or PELN-associated plant miRNAs reach recipient tissues at concentrations sufficient to produce biologically meaningful target repression.

### 2.3 Biodistribution, cellular uptake, and tissue targeting

The therapeutic potential of PELNs depends not only on their intrinsic bioactive cargos but also on their ability to reach target tissues and interact with recipient cells. Following administration, PELNs must overcome multiple biological barriers, including the gastrointestinal tract, vascular endothelium, extracellular matrix, and cellular membranes, before exerting biological effects. Therefore, understanding their biodistribution and tissue-targeting properties is fundamental for evaluating their translational potential in regenerative medicine.

Current studies suggest that the biodistribution of PELNs is influenced by multiple factors, including plant origin, vesicle size, membrane lipid composition, surface-associated molecules, route of administration, and cargo characteristics. Among these, the administration route appears to be particularly important. Oral delivery has been the most extensively investigated because many PELNs originate from edible plants and exhibit relatively high stability in the gastrointestinal environment (Guan et al., 2026). Experimental studies have demonstrated that orally administered ginger-derived and grape-derived nanovesicles can survive gastrointestinal transit, be internalized by intestinal epithelial cells and resident immune cells, and exert therapeutic effects in models of intestinal inflammation (Wang et al., 2014). These observations indicate that PELNs possess favorable stability and bioavailability compared with many synthetic nanocarriers.

Beyond the gastrointestinal tract, increasing evidence suggests that certain PELNs can reach extraintestinal tissues under specific experimental conditions. For example, engineered Panax notoginseng-derived nanovesicles carrying therapeutic miR-124 have been shown to cross the blood–brain barrier and improve neurological recovery after ischemic stroke (Li et al., 2023). Similarly, polydopamine-coated apple-derived nanovesicles exhibited enhanced accumulation within ischemic brain tissue, illustrating how surface engineering can substantially improve tissue targeting (Bai et al., 2026). These studies highlight the feasibility of directing PELNs toward otherwise difficult-to-access tissues, although such targeting generally relies on engineering strategies rather than the intrinsic properties of native vesicles.

Following tissue localization, PELNs are internalized by recipient cells through multiple uptake mechanisms, including endocytosis, macropinocytosis, phagocytosis, and membrane fusion. Recipient cells reported to internalize PELNs include macrophages, epithelial cells, endothelial cells, fibroblasts, and mesenchymal stromal cells. Cellular uptake enables the intracellular delivery of vesicular cargos, thereby initiating downstream signaling events associated with inflammation resolution, metabolic regulation, and tissue repair. However, the relative contribution of different uptake pathways appears to vary among cell types and remains incompletely understood (Li C. et al., 2025).

Membrane composition is likely to contribute to these interactions. Plant-derived vesicles contain phospholipids, glycolipids, and other lipid species that can affect vesicle stability and interactions with recipient-cell membranes. However, direct evidence that PELNs reorganize lipid rafts or receptor microdomains after uptake remains scarce. Proposed effects on TLR4 clustering, CXCR4 localization, or related membrane-signaling events should therefore be regarded as hypotheses derived largely from the broader EV literature rather than established properties of PELNs.

For musculoskeletal regeneration, direct evidence regarding the tissue-specific biodistribution of PELNs remains limited. Most available studies have demonstrated therapeutic efficacy in models of skeletal muscle, bone, cartilage, or peripheral nerve injury without systematically characterizing vesicle pharmacokinetics, tissue accumulation, or clearance profiles. Consequently, whether native PELNs possess intrinsic tropism for musculoskeletal tissues remains unknown. Future studies integrating quantitative fluorescence imaging, radionuclide labeling, single-vesicle tracking, and pharmacokinetic analyses will be essential to define tissue distribution, cellular uptake, and long-term biodistribution following systemic administration, thereby facilitating the rational development of PELN-based regenerative therapeutics.

Collectively, current evidence supports the notion that PELNs possess favorable biological compatibility and can effectively interact with mammalian recipient cells. Although their tissue-targeting mechanisms remain incompletely characterized, both native and engineered PELNs have demonstrated the capacity to overcome biological barriers and deliver functional cargos to recipient tissues. These biodistribution properties provide the prerequisite for the cross-kingdom biological activities discussed in the following section.

### 2.4 Cross-kingdom regulation of mammalian cells by PELNs

Following internalization by recipient cells, PELNs function as cross-kingdom biological regulators capable of influencing mammalian cellular behavior. Unlike conventional nanocarriers that primarily deliver exogenous therapeutic agents, PELNs contain endogenous bioactive cargos that interact with host cellular machinery and may modify multiple biological processes simultaneously. This ability to transmit biological information across kingdoms represents one of the distinguishing features of plant-derived nanovesicles (Liu et al., 2026).

Current evidence suggests that PELNs can influence mammalian cells at several interconnected levels. Following cellular uptake, vesicular cargos, including RNAs, proteins, lipids, and phytochemicals, may participate in the regulation of gene expression, intracellular signaling, and metabolic adaptation (Li C. et al., 2025). Rather than acting through a single molecular target, these cargos appear to function cooperatively, allowing PELNs to modulate complex cellular responses associated with tissue homeostasis and regeneration (Langellotto et al., 2025).

From the perspective of regenerative medicine, the biological significance of PELNs lies in their capacity to reshape cellular states rather than simply delivering isolated bioactive molecules. By coordinating multiple regulatory processes, PELNs may facilitate the transition of injured cells from stress-associated dysfunction toward a regeneration-permissive state.

## 3 PELNs in musculoskeletal regeneration

Musculoskeletal regeneration is a highly coordinated process involving restoration of cellular activity, reconstruction of tissue architecture, and re-establishment of a regeneration-permissive microenvironment. Following injury, aging, or chronic disease, these processes can be disrupted by persistent inflammation, oxidative stress, metabolic dysfunction, and impaired intercellular communication. Emerging studies have investigated PELNs in experimental models involving skeletal muscle, bone, cartilage, and peripheral nerve tissues, with reported effects on cell survival, differentiation, extracellular matrix remodeling, and other processes relevant to tissue regeneration. This section summarizes the tissue-specific evidence for PELNs in musculoskeletal repair, while distinguishing experimentally observed regenerative outcomes from broader mechanistic interpretations.

### 3.1 Skeletal muscle repair and regeneration

Skeletal muscle regeneration requires coordinated activation of myogenic progenitor cells and restoration of tissue homeostasis. Aging, injury, and chronic stress can impair this process through persistent inflammation, oxidative damage, and mitochondrial dysfunction, thereby contributing to progressive muscle loss and reduced regenerative capacity (Chargé and Rudnicki, 2004; Romanello and Sandri, 2021; Li W. et al., 2025).

Emerging evidence suggests that plant-derived nanovesicles may support skeletal muscle regeneration. A representative study showed that goji berry-derived nanovesicles (GqDNVs) attenuated dexamethasone-induced skeletal muscle atrophy and improved muscle-associated phenotypes (Zhou et al., 2024). Mechanistically, GqDNVs activated the AMPK/SIRT1/PGC-1α signaling pathway, which promoted mitochondrial biogenesis and improved energy metabolism, thereby counteracting glucocorticoid-induced muscle wasting.

Despite these encouraging findings, direct evidence for PELNs in skeletal muscle regeneration remains limited. In particular, further studies are needed to determine whether the reported molecular and morphological improvements translate into reproducible gains in muscle strength, endurance, or other performance-based outcomes, and whether similar effects extend to clinically relevant conditions.

### 3.2 Bone remodeling and skeletal regeneration

Bone regeneration is a complex and dynamic process that requires coordinated regulation of osteoblast-mediated bone formation, osteoclast-driven bone resorption, immune responses, and extracellular matrix (ECM) remodeling (Duda et al., 2023). Aging, chronic inflammation, metabolic imbalance, and metabolic dysfunction can disrupt these processes, resulting in impaired bone homeostasis and delayed skeletal repair.

Recent studies have reported osteogenesis-associated effects of PELNs derived from medicinal and edible plants. For example, Panax ginseng-derived nanovesicles (PGNVs) enhanced osteoblast-associated activity and bone-forming capacity in osteoporosis-related models (Lee et al., 2026). These findings provide direct experimental support for the potential application of plant-derived nanovesicles in modulating osteogenic responses.

Beyond direct regulation of osteoblast activity, PELNs may influence skeletal regeneration by improving the function of progenitor cells under unfavorable microenvironmental conditions. Inflammation-associated impairment of mesenchymal stromal cell (MSC) osteogenic differentiation represents a major limitation for bone repair. Recent evidence indicates that plant-derived fusion nanovesicles can rescue the inflammation-compromised osteogenic potential of periodontal ligament stem cells, suggesting that plant-derived vesicular systems may restore regenerative competence by reshaping inflammatory niches (Lin et al., 2025). Recent work has further identified nuclear FGF2-mediated regulation of rDNA chromatin organization and rRNA transcription as a mechanism controlling BMSC fate during skeletal regeneration (Zhang et al., 2025). These findings suggest that plant-derived vesicular systems may support osteogenic differentiation even under an unfavorable inflammatory microenvironment.

Additional evidence has been obtained in bone- and periodontal-regeneration models. Sea buckthorn-derived extracellular vesicles promoted osteogenic differentiation of bone marrow mesenchymal stromal cells and improved bone defect repair in experimental models (Zhao et al., 2025). Similarly, Paris polyphylla-derived extracellular vesicle-like particles enhanced periodontal ligament stem-cell proliferation, migration, and osteogenic differentiation and were associated with improved periodontal tissue regeneration (Huang et al., 2025). Together, these studies extend the available evidence beyond isolated osteogenic markers toward tissue-level regenerative outcomes, although the extent of functional or biomechanical recovery remains insufficiently characterized.

Collectively, current evidence indicates that PELNs may support skeletal regeneration through several interconnected processes, particularly through effects on osteoblast activity and progenitor-cell differentiation. However, the available studies remain heterogeneous in vesicle source, experimental model, and outcome assessment. Moreover, direct evidence linking these cellular and histological effects to restoration of bone mechanical properties or other functional outcomes remains limited.

### 3.3 Cartilage regeneration and joint repair

Cartilage degeneration represents a major challenge in musculoskeletal disorders due to the limited intrinsic regenerative capacity of cartilage tissue. Conditions such as osteoarthritis (OA) are characterized by progressive loss of extracellular matrix (ECM), impaired chondrocyte function, altered cellular metabolism, and chronic disruption of the joint microenvironment, ultimately leading to pain, reduced mobility, and functional impairment. Accordingly, regenerative strategies for cartilage disorders need to preserve chondrocyte activity and ECM homeostasis while limiting progressive tissue degeneration.

Emerging evidence suggests that PELNs may contribute to cartilage protection and regeneration by regulating chondrocyte behavior and maintaining ECM balance (Zuo et al., 2025). Among the available studies, grapefruit-derived plant extracellular vesicles (PDEVs) have been reported to regulate chondrocyte metabolic activity by enhancing anabolic responses while suppressing catabolic factors associated with cartilage degradation (Rashidi et al., 2025). In addition, these vesicles promoted chondrocyte migration, suggesting that PELNs may support cartilage repair by improving cellular mobility and regenerative capacity within damaged joint tissues.

However, compared with muscle and bone regeneration, current evidence regarding PELN-based cartilage repair remains relatively limited, and further studies are needed to clarify their tissue targeting mechanisms, cargo-specific functions, and long-term therapeutic effects in clinically relevant OA and cartilage injury models.

Collectively, current findings indicate that PELNs may support cartilage preservation and restoration of joint tissue homeostasis by regulating chondrocyte activity, extracellular matrix remodeling, and the local regenerative microenvironment. These multi-dimensional regulatory properties support their emerging role as promising nanobiological modulators for cartilage repair.

### 3.4 Peripheral nerve repair and neuromuscular regeneration

Peripheral nerve regeneration is essential for restoring neural input to skeletal muscle following nerve injury. Successful repair requires not only axonal regrowth but also coordinated interactions among Schwann cells, immune cells, and vascular components within the injured nerve microenvironment. Persistent oxidative stress, dysregulated immune responses, and inadequate vascular support can disrupt these processes and limit regenerative outcomes.

Emerging evidence suggests that PELNs may support peripheral nerve repair by modulating cellular responses within the injury microenvironment. In a recent study, Salvia miltiorrhiza-derived extracellular vesicles (SMEVs) were investigated in a peripheral nerve injury model and showed regenerative effects involving macrophage stress responses and angiogenic signaling (Huo et al., 2025). Following peripheral nerve injury, macrophages recruited to the injury site participate in myelin debris clearance and vascular remodeling, which are essential for Schwann cell migration and axonal regeneration. However, excessive uptake of myelin debris can induce lipid peroxidation and ferroptotic stress in macrophages, impairing their regenerative functions. SMEVs were shown to alleviate macrophage ferroptosis by enhancing the expression of ferroptosis-resistance regulators, including ferritin heavy chain 1 (FTH1) and glutathione peroxidase 4 (GPX4), while reducing the ferroptosis-associated factor ACSL4. This regulation decreased lipid peroxidation in macrophages and restored their pro-regenerative activity. SMEV treatment was also associated with enhanced macrophage-related angiogenic activity, including restoration of VEGFA expression and increased vascularization within the injured nerve microenvironment. These changes coincided with enhanced Schwann-cell migration and axonal regeneration, linking macrophage stress regulation with vascular and neural repair in this experimental model. These findings identify macrophage–vascular interactions as one potential mechanism through which plant-derived vesicles may support peripheral nerve regeneration.

Peripheral nerve regeneration is also relevant to musculoskeletal recovery because restoration of neural input is required to maintain skeletal muscle innervation and limit denervation-associated atrophy. However, direct evidence that PELNs improve integrated nerve–muscle functional recovery remains limited.

Collectively, available studies suggest that PELNs have regenerative potential across skeletal muscle, bone, cartilage, and peripheral nerve models, although the strength and type of evidence vary substantially among tissues. Current studies range from molecular and cellular observations to tissue-level regenerative outcomes, whereas direct functional assessments remain comparatively limited. Representative studies, experimental models, and reported outcomes are summarized in Table 1.

**TABLE 1:** Representative applications of plant-derived extracellular nanovesicles in musculoskeletal regeneration and functional rehabilitation.

Plant source	Experimental model	Evidence type	Target tissue	Key validated mechanism	Reported regenerative outcomes	Ref.
Gouqi (Lycium barbarum)	Dexamethasone-induced skeletal muscle atrophy	Both	Skeletal muscle	AMPK/SIRT1/PGC-1α activation	Reduced muscle atrophy; improved mitochondrial function	Zhou et al. (2024)
Panax ginseng	Osteoporosis-related experimental models	Both	Bone	Osteogenic differentiation	Enhanced bone formation	Lee et al. (2026)
Grapefruit	Chondrocyte/cartilage model	*In vitro*	Cartilage	Anabolic markers; catabolic enzymes	Improved cartilage repair	Rashidi et al. (2025)
Paris polyphylla	PDLSC and periodontal regeneration models	Both	Bone/periodontal tissue	Anti-inflammatory microenvironment	Accelerated alveolar bone regeneration	Huang et al. (2025)
Sea buckthorn	BMSC culture and murine femoral bone-defect model	Both	Bone	miRNA-mediated osteogenesis	Improved bone regeneration	Zhao et al. (2025)
Salvia miltiorrhiza	Cellular models and peripheral nerve injury	Both	Peripheral nerve	FTH1/GPX4/ACSL4/VEGFA	Enhanced nerve regeneration and functional recovery	Huo et al. (2025)
Turmeric	Inflammatory bone-loss models	Both	Bone–immune interface	NET inhibition; immune remodeling	Attenuated inflammatory bone destruction	Wang et al. (2026)

## 4 Mechanistic basis of PELN-mediated musculoskeletal regeneration

Musculoskeletal regeneration depends not only on activation of tissue-specific repair programs but also on the ability of injured cells and their surrounding microenvironment to resolve persistent stress-associated states. Chronic inflammation, oxidative imbalance, mitochondrial dysfunction, and metabolic disturbance can impair progenitor-cell activity and intercellular communication, thereby limiting regenerative capacity.

Across different experimental models, PELNs have been associated with changes in inflammatory signaling, redox balance, mitochondrial activity, and cellular metabolism. Although the underlying mechanisms vary among plant sources and experimental systems, these observations suggest that PELNs may influence interconnected stress-response pathways relevant to tissue regeneration. The following sections therefore focus on mechanistic processes supported by experimental evidence while distinguishing direct observations from mechanisms inferred across different tissue contexts.

### 4.1 Regulation of inflammatory stress and oxidative stress

Inflammatory activation and oxidative stress are closely interconnected during tissue injury (Kushioka et al., 2023). Persistent activation of inflammatory pathways, including NF-κB and inflammasome signaling, can amplify reactive oxygen species (ROS) production, whereas excessive ROS and lipid peroxidation can further sustain inflammatory responses and impair progenitor-cell function. Failure to resolve this inflammatory–redox imbalance can therefore create a microenvironment unfavorable for tissue regeneration.

PELNs have shown anti-inflammatory activity in several experimental systems, although much of the mechanistic evidence originates outside the musculoskeletal field. For example, Allium tuberosum-derived exosome-like nanoparticles (A-ELNs) reduced nitric oxide production and inflammatory cytokine expression in LPS-stimulated microglial models, accompanied by increased heme oxygenase-1 (HO-1) and decreased inducible nitric oxide synthase (iNOS) expression (Ishida et al., 2023). Although these findings were obtained in neural inflammatory models, they provide mechanistic evidence that plant-derived nanovesicles can modulate cellular inflammatory and redox responses.

Within a musculoskeletal-related context, the SMEV study discussed in Section 3.4 further links redox regulation to immune-cell function: attenuation of macrophage lipid peroxidation and ferroptotic stress was associated with preservation of pro-regenerative macrophage activity and angiogenic signaling (Huo et al., 2025). A similar coupling between redox regulation and immune responses has been observed in other tissue models. Ginkgo biloba-derived extracellular vesicles with reported peroxidase-like activity reduced ROS accumulation and inflammatory injury while being associated with M2 macrophage polarization and improved endothelial barrier function (Zhang Y. et al., 2026). Although this evidence was obtained outside the musculoskeletal system, it supports a potential mechanistic link between PELN-associated redox regulation and remodeling of inflammatory cellular states.

PELNs may also influence stress-induced cellular senescence. Artemisia argyi-derived nanovesicles (A-NVs) reduced intracellular and mitochondrial ROS and attenuated oxidative stress-induced senescence in human endometrial stromal cells (Zheng et al., 2026). Because this evidence derives from a non-musculoskeletal stromal-cell model, its relevance to musculoskeletal progenitor-cell aging remains inferential; nevertheless, it provides a rationale for investigating whether similar redox-sensitive responses occur in musculoskeletal regenerative cells.

Furthermore, skeletal regeneration requires substantial metabolic adaptation to support osteoblast differentiation and matrix synthesis. Mitochondrial dysfunction and impaired energy metabolism have been increasingly recognized as contributors to defective bone formation (Wang et al., 2023). Through their ability to influence cellular metabolism and stress adaptation, PELNs may help restore the energetic requirements of bone-forming cells and promote a regenerative phenotype.

Collectively, available evidence suggests that modulation of inflammatory and redox states is one recurring component of PELN-associated biological activity. However, direct demonstration of these mechanisms in musculoskeletal cells and tissues remains limited for several PELN systems. Because redox balance is closely coupled to mitochondrial function and cellular metabolism, these processes are considered together in the following section.

### 4.2 Mitochondrial homeostasis and metabolic adaptation

Mitochondria are central to cellular energy metabolism, redox balance, and stress adaptation. During tissue injury and chronic degeneration, impaired mitochondrial function can disrupt ATP production, increase oxidative stress, and compromise the activity of regenerative cell populations. These changes are particularly relevant to metabolically active or stress-sensitive cells involved in musculoskeletal repair, including muscle cells and tissue-resident progenitors.

Direct evidence linking PELNs to mitochondrial regulation in musculoskeletal models remains limited. In a dexamethasone-induced skeletal muscle atrophy model, goji berry-derived nanovesicles (GqDNVs) activated the AMPK/SIRT1/PGC-1α pathway and were associated with enhanced mitochondrial biogenesis and improved energy metabolism (Zhou et al., 2024). This study provides direct support for mitochondrial metabolic regulation as one mechanism associated with PELN-mediated protection in skeletal muscle.

Additional evidence comes from non-musculoskeletal models. Momordica charantia-derived extracellular vesicle-like nanovesicles attenuated mitochondrial oxidative stress and restored mitochondrial membrane potential in irradiated cardiomyocytes, accompanied by improved cell survival (Cui et al., 2022). Similarly, *Artemisia argyi*-derived nanovesicles reduced mitochondrial ROS and attenuated oxidative stress-induced cellular senescence in human endometrial stromal cells, with changes associated with SIRT1/PGC-1α/Nrf2 signaling (Zheng et al., 2026). These studies demonstrate that plant-derived nanovesicles can influence mitochondrial responses under cellular stress, but their findings cannot be directly extrapolated to musculoskeletal regeneration.

Current evidence is therefore insufficient to define comprehensive “mitochondrial reprogramming” as a general mechanism of PELN action. Most studies assess selected mitochondrial-associated endpoints, such as ROS levels, membrane potential, mitochondrial biogenesis, or expression of metabolic regulators. Direct measurements of oxidative phosphorylation, respiratory capacity, mitochondrial dynamics, mitophagy, and metabolic flux remain scarce, particularly in musculoskeletal models. Whether these mitochondrial changes are causally required for PELN-mediated regenerative effects also remains largely unresolved.

### 4.3 Remodeling of the regenerative microenvironment

Tissue regeneration depends on more than the response of individual cell types. Immune cells, vascular cells, resident progenitors, and the extracellular matrix all contribute to the local conditions in which repair takes place. When these interactions are disturbed by injury, aging, or chronic disease, regeneration can be delayed or incomplete. PELNs may affect several parts of this local environment, although current studies rarely examine the regenerative niche as a whole.

In skeletal muscle, functional recovery depends on the restoration of satellite cell activity, mitochondrial metabolism, and communication between muscle fibers, immune cells, and vascular components. Recent studies have suggested that plant-derived nanovesicles may regulate muscle regeneration by influencing myogenic differentiation, inflammatory resolution, and metabolic adaptation (Zhou et al., 2024). These findings support the concept that PELNs may enhance muscle recovery by restoring cellular metabolic flexibility rather than simply stimulating proliferation.

In addition to directly regulating resident cells, PELNs may contribute to tissue repair by improving the overall regenerative microenvironment. Carob-derived nanovesicles (CbNVs) have recently been characterized as plant-derived nanovesicles with anti-inflammatory and wound-healing properties (Manzaneque-López et al., 2026). CbNVs attenuated excessive inflammatory activation by suppressing NF-κB signaling in LPS-stimulated macrophages and promoted wound closure in keratinocytes, suggesting that plant-derived vesicles can facilitate tissue repair through coordinated regulation of inflammatory responses and regenerative processes. Collectively, these findings support the concept that modulation of the local inflammatory microenvironment represents an important mechanism by which PELNs promote tissue regeneration.

Collectively, current evidence indicates that PELNs may promote musculoskeletal recovery through coordinated reconstruction of the regenerative microenvironment. Their effects involve regulation of progenitor cell activation, immune resolution, vascular remodeling, and metabolic adaptation across multiple tissues. Rather than acting as isolated therapeutic molecules, PELNs appear to function as cross-cellular communication regulators that facilitate the transition from injury-associated dysfunction toward tissue restoration and functional recovery. This systemic regulatory capacity provides a mechanistic basis for exploring PELNs as potential biological modulators in exercise adaptation and rehabilitation.

## 5 An immune–metabolic–mitochondrial framework for regenerative rehabilitation

The mechanisms discussed above do not form a single pathway that has been demonstrated across PELN systems. Rather, studies using different plant sources and experimental models point to several recurring processes, including inflammatory regulation, redox control, mitochondrial responses, and changes in cellular metabolism. These processes are biologically linked, but the extent to which PELNs regulate them as an integrated network remains unclear. Here, we use an immune–metabolic–mitochondrial framework to consider how these findings may relate to tissue regeneration and rehabilitation (Figure 2).

**FIGURE 2:**
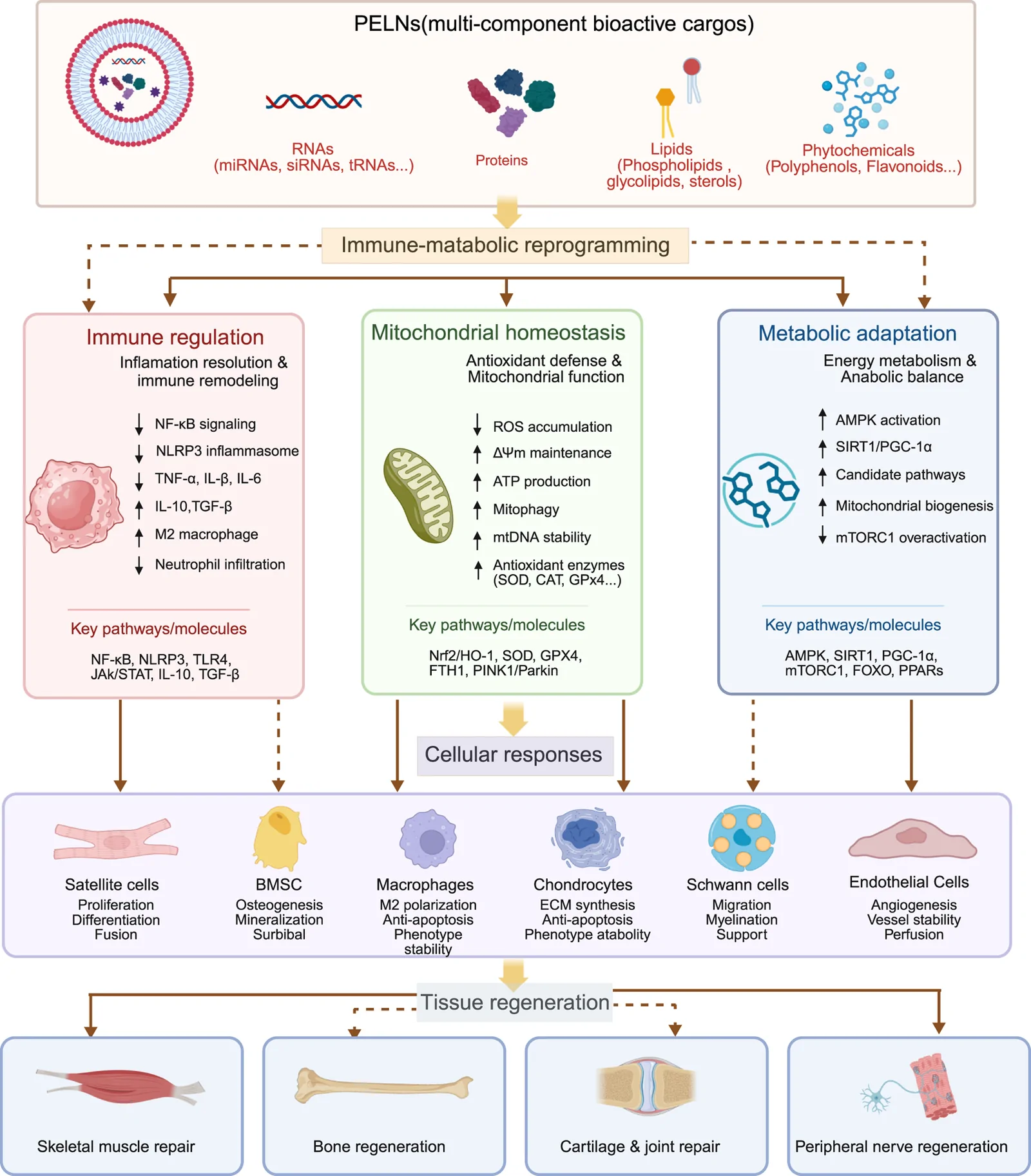
Proposed immune–metabolic–mitochondrial regulatory framework underlying PELN-mediated musculoskeletal regeneration and functional rehabilitation. PELNs carry diverse endogenous bioactive cargos, PELNs promote regenerative responses through three interconnected biological modules: (i) immune regulation; (ii) mitochondrial homeostasis; and (iii) metabolic adaptation. These coordinated responses influence the behavior of multiple regenerative cell populations, ultimately promoting skeletal muscle repair, bone regeneration, cartilage and joint repair, and peripheral nerve regeneration. Solid arrows indicate relationships directly supported by experimental evidence from PELN studies, whereas dashed arrows indicate proposed or indirect relationships inferred from non-musculoskeletal PELN models, broader extracellular vesicle research, or mechanistic integration across separate studies.

### 5.1 Immune–metabolic–mitochondrial crosstalk in tissue regeneration

Immune responses during tissue repair are closely tied to cellular metabolism and mitochondrial function. Macrophages provide a well-studied example. Inflammatory activation is often accompanied by greater reliance on glycolysis, whereas repair-associated states generally involve greater mitochondrial oxidative metabolism. These states are not fixed, however, and macrophage metabolism changes with local signals, nutrient availability, and redox conditions. Tissue repair therefore depends on the appropriate transition between inflammatory and reparative states rather than simply on suppression of inflammation.

PELNs can modify inflammatory responses, although direct evidence linking these effects to metabolic or mitochondrial changes is still limited. Ginger-derived exosome-like nanoparticles (GELNs), for example, inhibited NF-κB signaling in intestinal epithelial cells and suppressed NLRP3 inflammasome activation in macrophages (Chen et al., 2019; Yin et al., 2022). Grape-derived exosome-like nanoparticles (GrELNs), in a different setting, interacted with intestinal Lgr5^+^ stem cells and promoted Wnt/β-catenin-associated epithelial regeneration in DSS-induced colitis (Ju et al., 2013). These studies show that plant-derived nanovesicles can act on both inflammatory and regenerative cell populations, but they do not establish that the two effects are metabolically coupled.

Bone repair is also closely controlled by immune–bone interactions. Macrophages and other immune populations participate in different stages of skeletal healing by regulating inflammatory resolution and osteogenic signaling (Gou et al., 2024). Given the emerging immunomodulatory properties of PELNs, these vesicles may facilitate bone regeneration by coordinating immune responses with osteoblast activity and tissue remodeling.

### 5.2 Vesicle-mediated signaling in exercise adaptation and regenerative rehabilitation

Exercise provides a relevant physiological setting in which extracellular vesicle signaling has been studied in some detail. Acute physical activity increases the release of circulating extracellular vesicles (Frühbeis et al., 2015), and exercise-responsive vesicles carry proteins that may participate in communication between metabolically active tissues (Whitham et al., 2018). Vesicle release is therefore part of the systemic response to exercise, alongside changes in metabolism, inflammation, and tissue stress.

This does not mean that exercise-derived mammalian EVs and PELNs are functionally equivalent. They differ in origin, composition, and biological context, and evidence from exercise EVs cannot be used as direct evidence for PELN activity. The connection is instead based on the processes they may influence. As discussed above, PELNs have been reported to affect inflammatory signaling, redox balance, mitochondrial function, and regenerative cell responses. Many of these processes are also involved in tissue adaptation to exercise and in recovery after musculoskeletal injury. PELNs could therefore be relevant to regenerative rehabilitation, but this possibility remains largely unexplored.

A major gap is the lack of studies that directly test PELNs in exercise or rehabilitation settings. Most available work has focused on molecular, cellular, or histological outcomes in disease and injury models. Whether PELN treatment can improve muscle strength, endurance, gait, neuromuscular function, joint mobility, or recovery after exercise has rarely been examined. It is also unknown whether PELNs interact with exercise-induced signaling or simply produce parallel effects on some of the same biological processes. Addressing these questions will require experimental designs that combine PELN treatment with defined exercise or rehabilitation protocols and include functional endpoints alongside molecular and tissue-level measurements.

### 5.3 Mechanistic limitations and translational perspectives

The framework outlined above should be interpreted with several limitations in mind. PELNs from different plant species vary substantially in composition, and most have been studied in different cell types, disease models, and experimental settings. Findings obtained with one vesicle preparation therefore cannot be assumed to apply to PELNs as a whole. This heterogeneity also makes it difficult to determine whether the immune, metabolic, and mitochondrial effects described across studies reflect a shared mode of action or distinct properties of individual PELN systems.

Another unresolved issue is the link between vesicle cargo and biological activity. Many studies identify RNAs, proteins, lipids, or phytochemicals in PELNs and report changes in downstream signaling after treatment, but relatively few directly show that a specific cargo is responsible for the observed effect. Loss-of-function experiments, cargo depletion, and rescue approaches remain uncommon. The same caution applies to mechanisms inferred from non-musculoskeletal models. Such studies can point to pathways worth testing, but they cannot establish that the same mechanisms operate during muscle, bone, cartilage, or peripheral nerve regeneration.

Most importantly, the immune–metabolic–mitochondrial network proposed here has not been demonstrated as a complete pathway within a single PELN system. Current studies usually examine selected parts of this network, such as inflammatory signaling, mitochondrial stress, or metabolic regulation. Establishing causal links among these processes will require experiments that measure them in parallel and test whether disrupting one component alters the others and changes regenerative outcomes. Until then, the framework in Figure 2 is best viewed as a model that organizes the available evidence and defines questions for future study, rather than a fully established mechanism of PELN action.

## 6 Engineering PELNs for precision regenerative rehabilitation

Although native PELNs possess intrinsic regenerative and immunomodulatory activities, their clinical application remains constrained by limited tissue specificity, variable cargo composition, rapid clearance, and insufficient control over biodistribution. Recent advances in nanotechnology have therefore shifted the development of PELNs from naturally occurring biological vesicles toward programmable regenerative nanoplatforms. Rather than serving solely as passive delivery vehicles, engineered PELNs are increasingly designed to integrate targeting capability, therapeutic cargo loading, imaging guidance, and biomaterial-assisted delivery, thereby enabling precision rehabilitation strategies for musculoskeletal disorders.

Current engineering approaches can be broadly classified into three complementary strategies: surface engineering, functional hybridization, and biomaterial-assisted delivery (Table 2).

**TABLE 2:** Engineering strategies of PELNs for regenerative rehabilitation.

Engineering strategy	Representative modification	Evidence status in PELNs	Functional improvement	Representative application	Ref.
Surface engineering	PDA coating	Demonstrated	ROS scavenging; BBB targeting	Ischemic stroke	Bai et al. (2026)
Surface engineering	DSPE-PEG-RVG modification	Demonstrated	Enhanced BBB penetration and brain enrichment	Parkinson’s disease	Xu et al. (2024)
Ligand modification	Aptamer conjugation	Demonstrated	Enhanced cellular uptake and target selectivity	Brain/tumor-targeted delivery	Moon et al. (2023)
Cargo engineering	miRNA loading	Emerging	Delivery of regulatory RNA cargo	Gene regulation/targeted delivery	Shkryl et al. (2026)
Cargo engineering	Dexamethasone loading	Demonstrated	Enhanced anti-inflammatory activity	Neuroinflammation	Ishida et al. (2023)
Hybrid nanovesicles	Fusion membrane	Demonstrated	ROS-responsive delivery	Transplant rejection	Lu et al. (2024)

### 6.1 Surface engineering for tissue targeting

One major limitation of native PELNs is their relatively limited tissue specificity following systemic administration. Surface engineering aims to enhance cellular recognition, prolong circulation time, and improve accumulation within injured tissues by modifying vesicle membranes with functional molecules.

Among currently reported strategies, polydopamine (PDA) coating represents one of the most successful examples. PDA possesses excellent biocompatibility, antioxidant activity, and abundant reactive groups for secondary modification. A representative study engineered apple-derived extracellular nanovesicles coated with PDA and chelated with gadolinium (PDA@ELNs/Gd) for ischemic stroke therapy (Bai et al., 2026). The PDA coating improved vesicle stability and ROS-scavenging capability, while gadolinium enabled MRI visualization. This multifunctional platform exhibited enhanced blood–brain barrier penetration, promoted microglial M2 polarization, reduced infarct volume, and simultaneously enabled imaging-guided therapy, demonstrating the feasibility of integrating diagnosis and treatment into a single plant-derived nanoplatform.

Surface functionalization has also been explored through PEGylation, which improves colloidal stability, reduces nonspecific clearance, and prolongs systemic circulation. Although PEG-modified PELNs remain relatively limited compared with mammalian EVs, PEGylation represents a promising strategy for improving pharmacokinetic behavior in future rehabilitation applications.

Another emerging approach involves ligand-mediated targeting, in which peptides, antibodies, aptamers, or small molecules are conjugated onto vesicle membranes to achieve selective delivery toward injured muscle, bone, cartilage, or nervous tissues. Although this strategy has been extensively investigated in mammalian extracellular vesicles, its application to PELNs is still in its infancy and represents an important future direction for precision regenerative medicine.

### 6.2 Functional hybrid nanovesicles

Beyond membrane modification, increasing attention has focused on integrating PELNs with inorganic nanomaterials or therapeutic cargos to generate multifunctional hybrid systems.

One representative example is the previously mentioned PDA@ELNs/Gd platform, which combines antioxidant therapy with MRI-guided diagnosis, demonstrating that PELNs can simultaneously function as therapeutic and imaging nanocarriers.

Recently, ROS-responsive fusion extracellular nanovesicles have been developed by combining plant-derived nanovesicles with engineered membrane systems. These hybrid vesicles exhibited inflammation-responsive drug release and significantly alleviated acute transplant rejection through ROS-triggered activation, illustrating how environmental responsiveness can enhance therapeutic precision.

Another rapidly developing strategy involves incorporation of metal-based nanomaterials, including ZnO nanoparticles, Fe_3_O_4_ magnetic nanoparticles, and other bioactive inorganic components. These materials provide additional functionalities such as magnetic targeting, antibacterial activity, osteogenic stimulation, or imaging contrast. Although their application in PELNs for musculoskeletal rehabilitation remains limited, these multifunctional hybrid systems have demonstrated considerable promise in regenerative nanomedicine.

Functional cargo loading also represents an important engineering direction. PELNs have been successfully loaded with therapeutic molecules including dexamethasone, microRNA-124, and small interfering RNAs, thereby combining the intrinsic biological activity of plant vesicles with exogenous therapeutic cargos. For example, dexamethasone-loaded Allium tuberosum-derived nanovesicles (Dex-A-ELNs) exhibited stronger suppression of microglial inflammation than either free dexamethasone or native vesicles (Ishida et al., 2023), while erythrocyte membrane-fused Panax notoginseng-derived nanovesicles markedly prolonged circulation time and enhanced miR-124 delivery across the blood–brain barrier in ischemic stroke models (Li S. et al., 2025).

### 6.3 Biomaterial-assisted delivery for regenerative rehabilitation

While systemic administration offers broad tissue distribution, localized regenerative rehabilitation often requires prolonged retention of therapeutic vesicles within injured tissues. Biomaterial-assisted delivery therefore represents an increasingly important strategy for enhancing PELN efficacy.

Hydrogels provide an attractive platform by enabling sustained release, protecting vesicles from degradation, and maintaining high local concentrations at injury sites. Hydrogel encapsulation has been widely investigated for mammalian extracellular vesicles and is expected to substantially improve PELN retention in skeletal muscle, cartilage defects, and bone injuries. Similarly, three-dimensional scaffolds can combine mechanical support with biological signaling. Incorporation of PELNs into porous scaffolds may facilitate stem cell recruitment, extracellular matrix remodeling, and vascular ingrowth during bone regeneration.

Peripheral nerve repair represents another promising direction for biomaterial-assisted PELN delivery. Advanced nerve guidance conduits, including polycaprolactone (PCL)-based conduits, conductive scaffolds, and carbon quantum dot (CQD)-modified biomaterials, have demonstrated excellent capacity to support axonal guidance, Schwann cell migration, angiogenesis, and functional nerve regeneration (Zhang and Ma, 2024). Although direct integration of PELNs into these platforms has not yet been extensively explored, the intrinsic immunomodulatory and pro-regenerative properties of PELNs suggest that they could serve as bioactive cargos to further optimize the regenerative microenvironment and enhance peripheral nerve repair.

Collectively, engineering strategies are driving the evolution of PELNs from naturally occurring biological vesicles toward programmable regenerative nanoplatforms. Rather than merely improving drug delivery efficiency, next-generation engineered PELNs are increasingly designed to integrate targeted delivery, intelligent cargo release, immune regulation, metabolic reprogramming, and real-time imaging within a single therapeutic platform. Such multifunctional systems may provide new opportunities for precision rehabilitation following musculoskeletal injury, exercise-associated tissue damage, and degenerative disorders.

## 7 Clinical translation and challenges

Despite the promising regenerative effects observed in preclinical studies, the clinical translation of PELNs remains in its early stages. Their natural origin, low immunogenicity, and intrinsic bioactivity make them attractive candidates for regenerative rehabilitation; however, several challenges must be addressed before clinical application.

### 7.1 Standardization, isolation, and quality control

Standardization remains a practical barrier to comparing PELNs across studies. Unlike vesicles obtained from cultured mammalian cells, plant-derived preparations start from highly variable biological materials. Plant species, cultivar, tissue type, maturity, growing conditions, storage, and sample processing can all affect the composition of the starting material (Pinedo et al., 2021; Shu et al., 2025). Mechanical disruption during juice extraction or tissue homogenization adds another source of variation because intracellular membranes and other plant-derived particles may enter the vesicle-containing fraction. As a result, two preparations described under the same general term may differ substantially in particle composition and purity.

Isolation methods add a second layer of variation. Differential centrifugation and ultracentrifugation are widely used because they are relatively straightforward and suitable for processing plant-derived samples, but high-speed pelleting can co-isolate non-vesicular material and may promote particle aggregation. Density-gradient centrifugation improves separation on the basis of buoyant density and can produce cleaner vesicle-enriched fractions, although this comes at the cost of longer processing and, in some settings, lower recovery. Size-exclusion chromatography (SEC) separates particles according to size and can reduce contamination by soluble proteins without exposing vesicles to repeated high-speed centrifugation, but sample dilution and incomplete separation from similarly sized particles remain limitations. No single method provides optimal yield, purity, and scalability, and combinations of complementary separation approaches may therefore be preferable when highly purified preparations are required.

These methodological differences make particle yield alone an inadequate measure of preparation quality. At minimum, studies should report the source and amount of plant material, sample-processing procedures, centrifugation and filtration conditions, purification steps, particle concentration and size distribution, and measures of non-vesicular contamination. Morphological assessment and particle-sizing methods provide complementary information and are preferable to relying on a single characterization technique (Thieron et al., 2024; Welsh et al., 2024). Dose normalization also deserves more attention. PELN studies variously report particle number, protein concentration, or starting-material equivalents, making biological potency difficult to compare across preparations.

Quality control presents a particular challenge. The Minimal Information for Studies of Extracellular Vesicles (MISEV) recommendations provide a useful framework for documenting EV source, particle abundance, vesicle-associated components, and co-isolated non-vesicular material. However, these recommendations were developed largely around mammalian EV research and cannot be transferred directly to plant-derived preparations. Plant EV research still lacks a widely accepted panel of positive and negative markers that can be applied across plant species. Proteins such as TET8 and PEN1 have been investigated in specific plant EV populations, but their presence should not be interpreted as a universal marker for all PELNs. Until more robust plant-specific markers are established, claims about vesicle identity and purity are better supported by complementary physical, morphological, and molecular measurements rather than by a single marker.

Standardization also needs to extend beyond particle characterization. Preparations with similar size and particle concentration may still differ in lipid, RNA, protein, or phytochemical composition and therefore in biological activity. For translational studies, batch-to-batch consistency will eventually need to include both physicochemical attributes and biological potency. Functional assays linked to the intended application may be useful for this purpose, although these assays will need to be validated for individual PELN products rather than treated as universal quality-control measures.

### 7.2 Pharmacokinetics, safety, and delivery

Pharmacokinetic behavior is another major uncertainty. Although selected PELN systems have been detected in different organs or shown to cross specific biological barriers in preclinical models, quantitative data on absorption, circulation, tissue exposure, clearance, and dose–response relationships remain limited. This is particularly relevant to musculoskeletal applications, where the amount of administered PELNs that reaches muscle, bone, cartilage, or injured peripheral nerves is often unknown.

### 7.3 Clinical development and regulatory considerations

Clinical evidence remains far behind the rapidly expanding preclinical literature. Most PELN studies have been conducted in cell culture or animal models, and evidence from human intervention studies is still scarce. For regenerative rehabilitation in particular, clinical development will require more than demonstration of molecular or histological effects. Trials will need to define clinically meaningful outcomes, including physical function, pain, mobility, muscle performance, or other tissue-specific endpoints, depending on the intended indication.

Regulatory classification presents an additional challenge because PELNs combine features of naturally derived biological materials, nanoscale delivery systems, and, in some cases, engineered therapeutic products. Requirements for identity, purity, potency, safety, manufacturing consistency, and storage stability will need to be defined according to the final product and intended use. Engineered PELNs may face additional regulatory requirements because surface modification, exogenous cargo loading, or incorporation into biomaterials changes the composition of the original plant-derived preparation. Progress toward clinical use will therefore depend on closer alignment between biological characterization, scalable manufacturing, safety testing, and regulatory development.

### 7.4 Future perspectives

Future work should move from descriptive associations toward causal mechanisms. Although PELNs contain diverse RNAs, proteins, lipids, and phytochemicals, the components responsible for specific biological effects remain poorly defined. Cargo-depletion, loss-of-function, and rescue experiments could clarify cargo–function relationships and determine whether immune, metabolic, and mitochondrial responses are mechanistically linked within the same PELN system.

Mechanistic resolution at the tissue level is equally important. Combining single-cell or spatial profiling with quantitative biodistribution and cargo tracing could identify the cells that encounter and internalize PELNs *in vivo* and define how these responses change during repair. Such evidence is still limited in muscle, bone, cartilage, and peripheral nerve tissues.

Finally, molecular and histological effects need to be connected with functional outcomes. Studies incorporating defined exercise or rehabilitation protocols and measures of muscle performance, gait, joint function, biomechanics, or neuromuscular recovery would provide a stronger basis for evaluating PELNs in regenerative rehabilitation. Engineering strategies should be developed in parallel with reproducible manufacturing, safety assessment, and functional validation.

## 8 Discussion

PELNs have emerged as a promising class of naturally derived nanotherapeutics with broad potential for musculoskeletal regeneration and exercise rehabilitation. Accumulating evidence indicates that PELNs possess intrinsic biological activities beyond conventional cargo delivery and can influence processes relevant to tissue repair, including inflammatory signaling, redox balance, mitochondrial homeostasis, and regenerative microenvironment remodeling. Across skeletal muscle, bone, cartilage, and peripheral nerve models, these effects have been associated with favorable molecular, cellular, and histological outcomes. However, direct evidence for improvements in functional endpoints remains limited and varies substantially across experimental systems.

The available evidence does not support a single mechanism of PELN action. Instead, findings from different PELN systems point to interactions among inflammatory responses, cellular metabolism, and mitochondrial function. The immune–metabolic–mitochondrial framework proposed in this review provides a way to organize these observations, but it should not be interpreted as an established pathway shared by all PELNs. Most studies have examined individual components of this network, and direct evidence linking them within the same experimental system remains limited.

PELNs should also be considered within the broader context of stem-cell regulation and regenerative microenvironments. Current regenerative strategies increasingly recognize that tissue repair depends on both progenitor-cell competence and the surrounding immune environment (Wu et al., 2026). Although these studies do not provide direct evidence for PELN activity, they highlight regulatory levels that remain largely unexplored in PELN research, particularly the interaction between vesicle-mediated microenvironmental modulation and intrinsic stem-cell fate programs.

Translation of these findings will require greater consistency in PELN production and characterization, together with clearer pharmacokinetic, safety, and dose–response data. The evidence base also needs to progress from molecular and histological outcomes toward clinically relevant measures of recovery. For musculoskeletal rehabilitation, this includes muscle performance, gait, joint function, biomechanical properties, and neuromuscular recovery. Addressing these issues alongside mechanistic validation will be important for determining whether the regenerative effects observed experimentally can be translated into meaningful functional benefit.

## 9 Conclusion

PELNs are emerging as bioactive nanovesicles with potential applications in musculoskeletal regeneration. Current evidence indicates that they can influence inflammatory and redox responses, mitochondrial function, and regenerative cell activity, although the strength of evidence varies across tissues. The immune–metabolic–mitochondrial framework proposed in this review provides a basis for integrating these observations rather than defining a universal mechanism of PELN action. Further progress will depend on establishing causal mechanisms, standardized production and quality control, and clinically relevant functional outcomes. Addressing these gaps will be essential to determine the role of PELNs in regenerative rehabilitation and their potential for clinical translation.

## References

[B1] Alway S. E. Paez H. G. Pitzer C. R. (2023). The role of mitochondria in mediation of skeletal muscle repair. Muscles 2, 119–163. 10.3390/muscles2020011 40757564 PMC12225497

[B2] Arora N. K. Donath L. Owen P. J. Miller C. T. Saueressig T. Winter F. (2024). The impact of exercise prescription variables on intervention outcomes in musculoskeletal pain: an umbrella review of systematic reviews. Sports Med. 54, 711–725. 10.1007/s40279-023-01966-2 38093145 PMC10978700

[B3] Bai J. Liu W. Wang Z. Han B. Qi H. Sun J. (2026). MRI-guided diagnosis and dual-pathway neuroprotection in ischemic stroke via PDA-engineered apple exosome-like nanoparticles. Mat. Today, Bio 38, 103185. 10.1016/j.mtbio.2026.103185 PMC13194598 42182870

[B4] Barnett R. (2018). Osteoarthr. Lancet 391, 1985. 10.1016/S0140-6736(18)31064-X 29864015

[B5] Chargé S. B. P. Rudnicki M. A. (2004). Cellular and molecular regulation of muscle regeneration. Physiol. Rev. 84, 209–238. 10.1152/physrev.00019.2003 14715915

[B6] Chen X. Zhou Y. Yu J. (2019). Exosome-like nanoparticles from ginger rhizomes inhibited NLRP3 inflammasome activation. Mol. Pharm. 16, 2691–2699. 10.1021/acs.molpharmaceut.9b00246 31038962

[B7] Compston J. E. McClung M. R. Leslie W. D. (2019). Osteoporosis. Lancet 393, 364–376. 10.1016/S0140-6736(18)32112-3 30696576

[B8] Cruz-Jentoft A. J. Bahat G. Bauer J. Boirie Y. Bruyère O. Cederholm T. (2019). Sarcopenia: revised european consensus on definition and diagnosis. Age Ageing 48, 601. 10.1093/ageing/afz046 PMC6593317 31081853

[B9] Cui W.-W. Ye C. Wang K.-X. Yang X. Zhu P.-Y. Hu K. (2022). Momordica. Charantia-derived extracellular vesicles-like nanovesicles protect cardiomyocytes against radiation injury via attenuating DNA damage and mitochondria dysfunction. Front. Cardiovasc. Med. 9, 864188. 10.3389/fcvm.2022.864188 35509278 PMC9058095

[B10] Dad H. A. Gu T.-W. Zhu A.-Q. Huang L.-Q. Peng L.-H. (2021). Plant exosome-like nanovesicles: emerging therapeutics and drug delivery nanoplatforms. Mol. Ther. J. Am. Soc. Gene Ther. 29, 13–31. 10.1016/j.ymthe.2020.11.030 33278566 PMC7791080

[B11] Duda G. N. Geissler S. Checa S. Tsitsilonis S. Petersen A. Schmidt-Bleek K. (2023). The decisive early phase of bone regeneration. Nat. Rev. Rheumatol. 19, 78–95. 10.1038/s41584-022-00887-0 36624263

[B12] Eming S. A. Wynn T. A. Martin P. (2017). Inflammation and metabolism in tissue repair and regeneration. Science 356, 1026–1030. 10.1126/science.aam7928 28596335

[B13] Fan S.-J. Chen J.-Y. Tang C.-H. Zhao Q.-Y. Zhang J.-M. Qin Y.-C. (2022). Edible plant extracellular vesicles: an emerging tool for bioactives delivery. Front. Immunol. 13, 1028418. 10.3389/fimmu.2022.1028418 36569896 PMC9773994

[B14] Frontera W. R. Ochala J. (2015). Skeletal muscle: a brief review of structure and function. Calcif. Tissue Int. 96, 183–195. 10.1007/s00223-014-9915-y 25294644

[B15] Frühbeis C. Helmig S. Tug S. Simon P. Krämer-Albers E.-M. (2015). Physical exercise induces rapid release of small extracellular vesicles into the circulation. J. Extracell. Vesicles 4, 28239. 10.3402/jev.v4.28239 26142461 PMC4491306

[B16] Gao H. Pan L. Chen A. Xing X. Chen Q. Wang Y. (2026). Extracellular vesicles: key mediators of bioactive molecule transport in plant-microbe interactions. Mol. Plant Pathol. 27, e70314. 10.1111/mpp.70314 42433141 PMC13355006

[B17] Giraldo-Vallejo J. E. Cardona-Guzmán M. Á. Rodríguez-Alcivar E. J. Kočí J. Petro J. L. Kreider R. B. (2023). Nutritional strategies in the rehabilitation of musculoskeletal injuries in athletes: a systematic integrative review. Nutrients 15, 819. 10.3390/nu15040819 36839176 PMC9965375

[B18] Gou M. Wang H. Xie H. Song H. (2024). Macrophages in guided bone regeneration: potential roles and future directions. Front. Immunol. 15, 1396759. 10.3389/fimmu.2024.1396759 38736888 PMC11082316

[B19] Guan X. Zhu M. Zhu H. Wang Q. Chen J. Chen Y. (2026). Oral natural extracellular vesicles for biomedical applications: advances and clinical perspectives. J. Adv. Res. 83, 1105–1125. 10.1016/j.jare.2025.08.003 40783070 PMC13131399

[B20] Hao S. Yang H. Hu J. Luo L. Yuan Y. Liu L. (2024). Bioactive compounds and biological functions of medicinal plant-derived extracellular vesicles. Pharmacol. Res. 200, 107062. 10.1016/j.phrs.2024.107062 38211637

[B21] Harkins P. Burke E. Conway R. (2023). Musculoskeletal education in undergraduate medical curricula–a systematic review. Int. J. Rheum. Dis. 26, 211–224. 10.1111/1756-185X.14508 36502533 PMC10107471

[B22] Herrmann I. K. Wood M. J. A. Fuhrmann G. (2021). Extracellular vesicles as a next-generation drug delivery platform. Nat. Nanotechnol. 16, 748–759. 10.1038/s41565-021-00931-2 34211166

[B23] Hotamisligil G. S. (2006). Inflammation and metabolic disorders. Nature 444, 861–867. 10.1038/nature05485 17167474

[B24] Huang G. Liu A. Hu Y. Yang R. Dai Z. Meng W. (2025). Paris polyphylla var. yunnanensis leaf-derived extracellular vesicle-like particles enhance periodontal regeneration. Biomater. Res. 29, 291. 10.34133/bmr.0291 PMC12688648 41376817

[B25] Huang Y. Xu H. Bao S. Xiao B. (2026). Harnessing plant-derived extracellular vesicles for oral delivery: a dual role as natural therapeutics and engineered drug carriers. J. Control. Release Off. J. Control. Release Soc. 397, 115154. 10.1016/j.jconrel.2026.115154 42398832

[B26] Huo N. Xu G. Zhou H. Liu Y. Li S. Yang X. (2025). Nanovesicles synergistically regulate ferroptosis characteristic macrophage-induced angiogenesis via ferritin heavy chain 1/glutathione peroxidase 4 pathway to promote peripheral nerve regeneration. ACS Appl. Mat. Interfaces 17, 55876–55894. 10.1021/acsami.5c13694 40958080

[B27] Ishida T. Kawada K. Jobu K. Morisawa S. Kawazoe T. Nishimura S. (2023). Exosome-like nanoparticles derived from allium tuberosum prevent neuroinflammation in microglia-like cells. J. Pharm. Pharmacol. 75, 1322–1331. 10.1093/jpp/rgad062 37390476

[B28] Jo H. Y. Kang S. J. Kim G. Gwak S. Baek G. Rhee W. J. (2025). Plant-derived extracellular vesicles: current status and challenges for developing a new paradigm in therapeutics development. VIEW 6, 20240115. 10.1002/VIW.20240115

[B29] Ju S. Mu J. Dokland T. Zhuang X. Wang Q. Jiang H. (2013). Grape exosome-like nanoparticles induce intestinal stem cells and protect mice from DSS-induced colitis. Mol. Ther. 21, 1345–1357. 10.1038/mt.2013.64 23752315 PMC3702113

[B30] Kushioka J. Chow S. K.-H. Toya M. Tsubosaka M. Shen H. Gao Q. (2023). Bone regeneration in inflammation with aging and cell-based immunomodulatory therapy. Inflamm. Regen. 43, 29. 10.1186/s41232-023-00279-1 37231450 PMC10210469

[B31] Langellotto M. D. Rassu G. Serri C. Demartis S. Giunchedi P. Gavini E. (2025). Plant-derived extracellular vesicles: a synergetic combination of a drug delivery system and a source of natural bioactive compounds. Drug Deliv. Transl. Res. 15, 831–845. 10.1007/s13346-024-01698-4 39196501 PMC11782344

[B32] Lee S.-H. Kwon J.-H. Kim H.-W. Mun S. H. Yang Y. Kim J. (2026). Therapeutic potential of panax ginseng-derived nanovesicles in osteoporosis through enhanced osteoblast. J. Ginseng Res. 50, 101016. 10.1016/j.jgr.2026.101016 42112125 PMC13149911

[B33] Li S. Zhang R. Wang A. Li Y. Zhang M. Kim J. (2023). Panax notoginseng: derived exosome-like nanoparticles attenuate ischemia reperfusion injury via altering microglia polarization. J. Nanobiotechnol. 21, 416. 10.1186/s12951-023-02161-1 37946257 PMC10636993

[B34] Li C. Zeng A. Li L. Zhao W. (2025a). Emerging roles of plant-derived extracellular vesicles in biotherapeutics: advances, applications, and future perspectives. Adv. Biol. 9, e2500008. 10.1002/adbi.202500008 40197701

[B35] Li S. Wang A. Zhang R. Zhang M. Guo P. Chen B. (2025b). An erythrocyte membrane-fused plant-derived nanoparticles as a gene therapy vehicle for the treatment of CI/R injury. Asian J. Pharm. Sci. 20, 101089. 10.1016/j.ajps.2025.101089 41809261 PMC12547663

[B36] Li W. Chen M. Zhang L. (2025c). Muscle stem cell microenvironment and functions in muscle regeneration. Biomolecules 15, 765. 10.3390/biom15060765 40563407 PMC12190381

[B37] Lin J. Li M. Wang L. Lu X. Xu Q. Chen H. (2025). Plant and animal-derived fusion nanovesicles rescue inflammation-compromised osteogenic potential of periodontal ligament stem cells. Front. Cell. Dev. Biol. 13, 1512238. 10.3389/fcell.2025.1512238 40083664 PMC11903722

[B38] Liu W. Yin X. Zhang Z. Chou W. Xin Y. Zhang Y. (2026). Plant-derived exosome-like nanoparticles: mechanisms of cross-kingdom regulation and perspectives as natural drug carriers for disease treatment. Int. J. Nanomed. 21, 581774. 10.2147/IJN.S581774 41884278 PMC13012304

[B39] Lu X. Xu Z. Shu F. Wang Y. Han Y. Yang X. (2024). Reactive oxygen species responsive multifunctional fusion extracellular nanovesicles: prospective treatments for acute heart transplant rejection. Adv. Mat. 36, e2406758. 10.1002/adma.202406758 38949397

[B40] Lu C. Li D. Gao W. Sun X. Zhang Y. Yu Y. (2026). Plant-derived extracellular vesicles in skin and bone tissue engineering: current status, challenges, and future perspectives. Front. Bioeng. Biotechnol. 14, 1764724. 10.3389/fbioe.2026.1764724 41938063 PMC13044024

[B41] Manzaneque-López M. C. Sánchez-López C. M. Marcilla A. Pérez-Bermúdez P. Soler C. (2026). Isolation and functional characterization of carob-derived nanovesicles reveals anti-inflammatory and regenerative potential. Plants 15, 590. 10.3390/plants15040590 41754297 PMC12944457

[B42] Mitchell M. J. Billingsley M. M. Haley R. M. Wechsler M. E. Peppas N. A. Langer R. (2021). Engineering precision nanoparticles for drug delivery. Nat. Rev. Drug Discov. 20, 101–124. 10.1038/s41573-020-0090-8 33277608 PMC7717100

[B43] Moon K. Hur J. Kim K. P. Lee K. Kang J. Y. (2023). Surface-functionalizable plant-derived extracellular vesicles for targeted drug delivery carrier using grapefruit. Adv. Mat. Interfaces 10, 2300220. 10.1002/admi.202300220

[B44] Mu J. Zhuang X. Wang Q. Jiang H. Deng Z.-B. Wang B. (2014). Interspecies communication between plant and mouse gut host cells through edible plant derived exosome-like nanoparticles. Mol. Nutr. Food Res. 58, 1561–1573. 10.1002/mnfr.201300729 24842810 PMC4851829

[B45] Pinedo M. de la Canal L. de Marcos Lousa C. (2021). A call for rigor and standardization in plant extracellular vesicle research. J. Extracell. Vesicles 10, e12048. 10.1002/jev2.12048 33936567 PMC8077130

[B46] Rahman F. A. Quadrilatero J. (2021). Mitochondrial network remodeling: an important feature of myogenesis and skeletal muscle regeneration. Cell. Mol. Life Sci. CMLS 78, 4653–4675. 10.1007/s00018-021-03807-9 PMC11072563 33751143

[B47] Rashidi N. Liu C. Guillot P. V. Tamaddon M. (2025). Isolation, characterization, and *in vitro* cell studies of plant-based exosome-like nanovesicles for treatment of early osteoarthritis. Int. J. Mol. Sci. 26, 2211. 10.3390/ijms26052211 40076829 PMC11900001

[B48] Rawat B. Rajpoot Y. S. Saha S. Ciocan V.-C. Cristuta A.-M. Choudhary S. (2026). Is exercise enough? Evidence from controlled clinical trials on rehabilitation with and without adjunct modalities for musculoskeletal disorders. Life 16, 608. 10.3390/life16040608 42073419 PMC13117354

[B49] Romanello V. Sandri M. (2021). The connection between the dynamic remodeling of the mitochondrial network and the regulation of muscle mass. Cell. Mol. Life Sci. CMLS 78, 1305–1328. 10.1007/s00018-020-03662-0 33078210 PMC7904552

[B50] Schieber M. Chandel N. S. (2014). ROS function in redox signaling and oxidative stress. Curr. Biol. CB 24, R453–R462. 10.1016/j.cub.2014.03.034 24845678 PMC4055301

[B51] Seo K. Yoo J. H. Kim J. Min S. J. Heo D. N. Kwon I. K. (2023). Ginseng-derived exosome-like nanovesicles extracted by sucrose gradient ultracentrifugation to inhibit osteoclast differentiation. Nanoscale 15, 5798–5808. 10.1039/d2nr07018a 36857681

[B52] Shkryl Y. Tsydeneshieva Z. Gorpenchenko T. Kazarin V. Kudinova O. Bulgakov V. (2026). Artificial MicroRNA delivery and gene silencing via extracellular vesicles derived from molecularly modified tobacco. Protoplasma 263, 875–891. 10.1007/s00709-025-02130-5 41366159

[B53] Shu F. Hu Y. Sarsaiya S. Jin L. Yang X. Liu F. (2025). Plant-derived extracellular vesicles quality control: key process progress and future research directions. Discov. Nano 20, 233. 10.1186/s11671-025-04399-0 41400892 PMC12708502

[B54] Spinelli J. B. Haigis M. C. (2018). The multifaceted contributions of mitochondria to cellular metabolism. Nat. Cell. Biol. 20, 745–754. 10.1038/s41556-018-0124-1 29950572 PMC6541229

[B55] Thieron H. Krassini L. Kwon S. Fricke S. Nasfi S. Oberkofler L. (2024). Practical advice for extracellular vesicle isolation in plant-microbe interactions: concerns, considerations, and conclusions. J. Extracell. Vesicles 13, e70022. 10.1002/jev2.70022 39665314 PMC11635479

[B56] Tidball J. G. (2017). Regulation of muscle growth and regeneration by the immune system. Nat. Rev. Immunol. 17, 165–178. 10.1038/nri.2016.150 28163303 PMC5452982

[B57] Wang B. Zhuang X. Deng Z.-B. Jiang H. Mu J. Wang Q. (2014). Targeted drug delivery to intestinal macrophages by bioactive nanovesicles released from grapefruit. Mol. Ther. J. Am. Soc. Gene Ther. 22, 522–534. 10.1038/mt.2013.190 23939022 PMC3944329

[B58] Wang J. Zhang Y. Cao J. Wang Y. Anwar N. Zhang Z. (2023). The role of autophagy in bone metabolism and clinical significance. Autophagy 19, 2409–2427. 10.1080/15548627.2023.2186112 36858962 PMC10392742

[B59] Wang X. Xin C. Zhou Y. Sun T. (2024). Plant-derived vesicle-like nanoparticles: the next-generation drug delivery nanoplatforms. Pharmaceutics 16, 588. 10.3390/pharmaceutics16050588 38794248 PMC11125130

[B60] Wang M. Tuerhong K. Xie Y. Huang Q. Zhang Q. Liu K. (2026). Turmeric-derived nanovesicles reprogram pathological NETosis to mitigate inflammatory bone loss. J. Nanobiotechnol. 24, 813. 10.1186/s12951-026-04682-x 42310760 PMC13520241

[B61] Welsh J. A. Goberdhan D. C. I. O’Driscoll L. Buzas E. I. Blenkiron C. Bussolati B. (2024). Minimal information for studies of extracellular vesicles (MISEV2023): from basic to advanced approaches. J. Extracell. Vesicles 13, e12404. 10.1002/jev2.12404 38326288 PMC10850029

[B62] Whitham M. Parker B. L. Friedrichsen M. Hingst J. R. Hjorth M. Hughes W. E. (2018). Extracellular vesicles provide a means for tissue crosstalk during exercise. Cell. Metab. 27, 237–251.e4. 10.1016/j.cmet.2017.12.001 29320704

[B63] Wiklander O. P. B. Brennan M. Á. Lötvall J. Breakefield X. O. El Andaloussi S. (2019). Advances in therapeutic applications of extracellular vesicles. Sci. Transl. Med. 11, eaav8521. 10.1126/scitranslmed.aav8521 31092696 PMC7104415

[B64] Woith E. Fuhrmann G. Melzig M. F. (2019). Extracellular vesicles-connecting kingdoms. Int. J. Mol. Sci. 20, 5695. 10.3390/ijms20225695 31739393 PMC6888613

[B65] Wu X. Jin S. Pan Y. Zhen W. Yu S. Zhang Y. (2026). Immune-evasive stem cells: engineering tolerance and reprogramming microenvironments for regenerative therapy. Stem Cell. Res. Ther. 17, 190. 10.1186/s13287-026-04997-4 41937145 PMC13188328

[B66] Xu Y. Yan G. Zhao J. Ren Y. Xiao Q. Tan M. (2024). Plant-derived exosomes as cell homogeneous nanoplatforms for brain biomacromolecules delivery ameliorate mitochondrial dysfunction against parkinson’s disease. Nano Today 58, 102438. 10.1016/j.nantod.2024.102438

[B67] Yin H. Price F. Rudnicki M. A. (2013). Satellite cells and the muscle stem cell niche. Physiol. Rev. 93, 23–67. 10.1152/physrev.00043.2011 23303905 PMC4073943

[B68] Yin L. Yan L. Yu Q. Wang J. Liu C. Wang L. (2022). Characterization of the MicroRNA profile of ginger exosome-like nanoparticles and their anti-inflammatory effects in intestinal caco-2 cells. J. Agric. Food Chem. 70, 4725–4734. 10.1021/acs.jafc.1c07306 35261246

[B69] Yu J. Shen Y. Jia H. (2026). Plant-derived extracellular vesicles as bioactive nanoplatforms: biogenesis, therapeutic evidence, and translational challenges. Biochem. Biophys. Rep. 47, 102688. 10.1016/j.bbrep.2026.102688 42395865 PMC13324295

[B70] Zeng Y.-B. Deng X. Shen L.-S. Yang Y. Zhou X. Ye L. (2024). Advances in plant-derived extracellular vesicles: isolation, composition, and biological functions. Food Funct. 15, 11319–11341. 10.1039/d4fo04321a 39523827

[B71] Zhang Z. Ma M. (2024). Strategies to enhance the ability of nerve guidance conduits to promote directional nerve growth. Biomed. Eng. Online 23, 40. 10.1186/s12938-024-01233-z 38582838 PMC10998375

[B72] Zhang L. Hou D. Chen X. Li D. Zhu L. Zhang Y. (2012). Exogenous plant MIR168a specifically targets mammalian LDLRAP1: evidence of cross-kingdom regulation by microRNA. Cell. Res. 22, 107–126. 10.1038/cr.2011.158 21931358 PMC3351925

[B73] Zhang S. Xing M. Li B. (2019). Recent advances in musculoskeletal local drug delivery. Acta Biomater. 93, 135–151. 10.1016/j.actbio.2019.01.043 30685475 PMC6615977

[B74] Zhang H. Wang Z. Liu Z. Li X. Sun W. Zhen W. (2025). Nuclear FGF2 orchestrates phase separation-mediated rDNA chromatin architecture to control BMSCs cell fate. Bone Res. 13, 80. 10.1038/s41413-025-00451-y 40993129 PMC12460815

[B75] Zhang C. Wang Y. Jiang X. Wang D. Yuan Y. Li J. (2026a). M1 macrophage-targeted engineered ginseng stems and leaves-derived extracellular vesicles delivery system for alleviating rheumatoid arthritis. Regen. Biomater. 13, rbag078. 10.1093/rb/rbag078 PMC13213593 42211882

[B76] Zhang Y. Wu J. Hu X. Meng X. Guo Z. Guo Z. (2026b). Peroxidase-enriched extracellular vesicles from ginkgo biloba ameliorate acute lung injury via ROS scavenging, M2 macrophage polarization and barrier protection. ACS Appl. Mat. Interfaces 18, 29794–29809. 10.1021/acsami.6c07953 42178839

[B77] Zhao M. Chen X. Wang W. Li M. Zhang H. Zou X. (2025). Sea buckthorn-derived extracellular vesicles foster bone regeneration through aau-miR168-mediated pathways. Stem Cell. Res. Ther. 16, 281. 10.1186/s13287-025-04373-8 40462231 PMC12135495

[B78] Zheng X. Huang R. Liu Z. Liu T. Lin H. Yin L. (2026). Preparation of artemisia argyi-derived extracellular nanovesicles and their protective effects against oxidative stress-induced senescence in endometrial stromal cells. Bioeng. (Basel) 13, 256. 10.3390/bioengineering13030256 41899787 PMC13024543

[B79] Zhou X. Xu S. Zhang Z. Tang M. Meng Z. Peng Z. (2024). Gouqi-derived nanovesicles (GqDNVs) inhibited dexamethasone-induced muscle atrophy associating with AMPK/SIRT1/PGC1α signaling pathway. J. Nanobiotechnol. 22, 276. 10.1186/s12951-024-02563-9 38778385 PMC11112783

[B80] Zuo Y. Zhang J. Sun B. Wang X. Wang R. Tian S. (2025). A new perspective on regenerative medicine: plant-derived extracellular vesicles. Biomolecules 15, 1095. 10.3390/biom15081095 40867539 PMC12383368

